# Audit of Pediatric ENT Injuries

**Published:** 2011-09

**Authors:** S. K. Aremu, B. S. Alabi, S. Segun-Busari, W. Omotoso

**Affiliations:** *University of Ilorin Teaching Hospital, Ilorin, Nigeria*

**Keywords:** trauma, ear, nose, throat, mechanism, profile of injury in children

## Abstract

**Background:**

Ear, Nose and throat (ENT) injuries are relatively common in children. Sometimes they may result in disfigurement or dysfunction of the affected parts. This study was aimed at the pattern and mechanisms of ENT injuries encountered by children in Ilorin located in north central Nigeria. It is therefore our hope that the information will go a long way to assist other African countries as well in health care plans for children.

**Objectives:**

The study was aimed at determining the pattern and causes of ENT injuries in children in Ilorin.

**Methodology:**

This was a descriptive cross sectional, prospective study of all pediatrics patients aged 15 years and below who were managed between January 2005 and December 2009 (a period of 5 years) for ENT trauma in the accident & emergency unit, wards and clinics of University of Ilorin Teaching Hospital. Most of them were treated as out-patients; a few of them needed admission for some procedures. Patients with injuries to other areas than ENT were not included in this study. The data that were analyzed included the age, sex, presenting complaints, duration of symptoms prior to presentation, diagnoses, treatments and complications.

**Results:**

Two hundred and seventy-one children were studied, of which 168 (62.0%) were males and 103 (38.0%) were females. Most of the children, 97 (35.8%), were between age group of 6-10 years, 81 (29.9%) aged 5 years and below, while the rest were 11-15 years of age. Injuries due to foreign bodies in Ear, Nose and Throat accounted for 80 (29.5%) of the causes. Falls accounted for 60 (24.4%) of cases while Road Traffic Injuries (RTI), 42 (15.5%) of cases. Bleeding was the commonest presenting symptom. Most of the children, 97 (35.8%) presented within 1 hour of injury while the least, 45 (16.6%), presented more than 8 hours after the injury. 132 (48.7%) had injuries in the Ear, 117 (43.2%) in the Nose and 22 (8.1%) in the Throat.

**Conclusion:**

ENT injuries in pediatrics are relatively common in our environment with injuries from FB insertion/ingestion being the commonest. This is closely followed by falls. Several disabilities and morbidity may result from the trauma like anosmia, facial palsy and permanently depressed nose. These have impact on psychology of the child. For these reason close monitoring of children by parents and care-givers cannot be overemphasized. Also these injuries are avoidable causes of death and disability in children.

## INTRODUCTION

Injuries to Ear Nose and Throat (ENT) in children are considered as an inevitable part of children experience. The mechanisms and causes of ENT injuries in children are different from those in adults (1). These are avoidable causes of death and disability in children (2). Injuries are more common cause of mortality and morbidity in adults (3). Similarly the types of injuries in developing countries are different from those in well developed and industrialized countries (4). Injuries may affect different parts of the body. There is scarcity of international literature on ENT injuries in young adults and children. We present this study to provide us an insight into the profiles of injuries in children and their mechanisms in Ilorin.

## PATIENTS AND METHODS

This was a descriptive cross sectional, prospective study of all pediatrics patients aged 15 years and below who were managed between January 2005 and December 2009 for ENT trauma in the accident & emergency unit, wards and clinics of University of Ilorin Teaching Hospital. The hospital is located in Ilorin, the Kwara state capital in the middle belt zone of Nigeria, sub-serverving about eight states namely Kwara, Kogi, Niger, Oyo, Osun, Ekiti, Lagos and part of Kebbi states. Department of Ear, Nose and Throat is one of the hospital departments with services covering the above mentioned zones in terms of patients’ coverage.

Two hundred and seventy-one pediatric patients were enrolled in the study. Patients with injuries to other areas than ENT were not included in this study. Most of the subjects were treated as out-patients, a few of them needed admission for some procedures. The data that were analyzed included the age, sex, presenting complaints; duration of symptoms prior to presentation, diagnoses, treatments and complications. Analysis was done with SPSS version 11.0 computer software.

## RESULTS

The total number of patients included in this study was 271. All patients were treated successfully. Of all patients 168 (62.0%) were males and 103 (38.0%) were females. Most of the children, 97 (35.8%), falls between age group of 6-10 years, 81 (29.9%) aged 5 years and below, while the rest were 11-15 years of age. Most of the patients were from the urban areas. 80 (29.5%) of the injuries were due to foreign bodies in Ear, Nose and Throat. Falls accounted for 60 (24.4%) of cases while Road Traffic Injuries (RTI), 42 (15.5%) of cases (Table 1). In the Ear and Nose the most common mode of presentation is Foreign Bodies (FB) insertion, while bleeding from Oral cavity was the commonest in the Throat. Others were as shown in Table 2. Most of the children, 97 (35.8%) presented within 1 hour of injury while the least, 45 (16.6%), presented more than 8 hours (Table 3). 132 (48.7%) had injuries in the Ear, 117 (43.2%) in the Nose and 22 (8.1%) in the Throat (Table 4). The residual complications included perforated tympanic membrane, facial scar, pinna deformity and external nasal deformity.

**Table 1:** Causes of injuries in relation to age group of children

CAUSES	0-5 yrs	6-10 yrs	11-15 yrs	TOTAL (no. & %)

ENT Foreign Body Injuries	30	25	25	80 (29.5)
Falls	30	21	15	60 (24.4)
RTI	13	14	15	42 (15.5)
Pointed object	4	8	5	17 (6.3)
Blunt Trauma	0	19	21	40 (14.8)
Knife Injury	0	3	2	5 (1.8)
Animal Injury	1	0	2	3 (1.1)
Burns (flames &caustics)	2	4	6	12 (4.4)
Machine Injury	1	3	2	6 (2.2)
TOTAL (no. & %)	81 (29.9)	97 (35.8)	93 (34.3)	271 (100)

**Table 2:** Presenting Symptoms

Ear		Nose		Throat	
FB Insertion	39	FB Insertion	37	Bleeding from Oral cavity	7
Ear Pain	26	Epistaxis	35	Trismus/Dysphagia	5
Bleeding	20	Rhinorrhoea	19	FB aspiration/Ingestion	4
Otorrhoea	19	Facial swelling	13	Dyspnoea/Stridor	3
Hearing Loss	16	Nasal laceration/fracture	13	Corrosives Ingestion	3
Pinna laceration/avulsion	12				

**Table 3:** Duration of symptoms

Duration(hrs)	No of Patients	Percentage (%)

0-1	97	35.8
2-4	73	26.9
5-7	56	20.7
>8	45	16.6

**Table 4:** ENT distribution of injuries

Region	No of Patients	Percentage (%)

Ear	132	48.7
Nose	117	43.2
Throat	22	8.1
Total	271	100

## DISCUSSION

ENT injuries pediatric age groups are not very uncommon. A study showed that more than 80% of the patients were from rural areas (5). But in our own study most of them were from urban areas. Males dominated the study with a male to female ratio of 1.6:1.0. The high percentage of males seems to be due to more outdoor activities than girls. The boys are more adventurous and indulge more in cycling, jumping, climbing and other activities. Similar findings were reported by Ebong (6). Most of the patients, 97 (35.8%), were in 6-10 years of life.

Foreign body related injuries constituted the commonest cases seen in our study. This group of patient presented in ways dictated by the site of FB lodgment. In the ear, they presented with bleeding, otalgia, impaired hearing or otorrhoea. In the nose, they presented with nasal discharge of thick and offensive mucus. The presentation was more dramatic in the airway with presentation of dyspnoea and stridor. All the foreign bodies in the ear and nose were successfully removed, that is 100% treatment success rate. The patients with foreign bodies in the throat were less dramatic, presenting with dysphagia, odynophagia as the common features (7).

Analysis of injury sites in our study showed that nose was the second commonest site of injury, 117 (43.2%), which manifested as epistaxis, facial swelling, rhinorrhoea and nasal laceration/fracture. A similar finding was observed in previous studies by Ghai *et al* (8). The nasal trauma was commonly due to foreign body, RTI and fall. Road side accidents were commonly encountered in older children. This is because of more outdoor activities and road side cycling in grown up children. The severity of these injuries may be underestimated unless intranasal examination is performed (9). Ear involvement with injury to the pinna was commonly found in road side accidents. There were cases of associated haemotympanum and tympanic membrane perforations. The events responsible for falls included, falls from trees, stairs, kite flying and from the walls or roof tops and working at agricultural fields. Below the age of 5 years fall from the cot, bed, table or chair were noted. Cases of nasal soft tissue laceration/avulsion were treated with suturing and nasal fractures were stented after realignment. Those who presented late with disfigurement had rhinoplasty done. All patients were covered with antibiotics to prevent infection. The follow up periods ranged between 3-6 months as the case warranted. Pinna lacerations were sutured and complete healing achieved. A case of complete pinna avulsion later had prosthesis replacement. All the 3 cases of corrosive ingestion.were managed conservatively, as they presented early, with gentle passage of nasogastric tube, antibiotic, anti-inflammatory drugs and antacids. A barium swallow done after 3 weeks showed that none of them had oesophageal stricture.

## CONCLUSION

ENT injuries in pediatrics are relatively common in our environment with injuries from FB insertion/ ingestion being the commonest. This is closely followed by falls. Several disabilities and morbidity may result from the trauma like anosmia, facial palsy and permanently depressed nose. These have impact on psychology of the child. Early presentation and prompt treatment will prevent such complications. Also these injuries are avoidable causes of death and disability if children are closely monitored by parents and care-givers.
